# 

**Published:** 1863-06

**Authors:** 


### CONTENTS.

ORIGINAL ESSAYS AND COMMUNICATIONS.

Development of Dental Tissues. By W. H. A ........ 237
Amalgam Fillings. By Geo. Foote, M. D........ 241

PROCEEDINGS OF SOCIETIES.

Meeting of Dentists in Syracuse, N. Y........ 245

CORRESPONDENCE.

The American Dental Association,........ 247
Letter from "J,"........ 248
Letter from St. Louis,........ 250

SELECTIONS.

Gold Dust and Iron Filings as an Antidote for Corrosive Sublimate,........ 251
On the use of Glycerine in Surgery and Medicine........ 255
Practical Hints,........ 259
Influence of the Sun's Rays in the production of Organic Matter,........ 261
Amalgam,........ 264
Disinfectants and their Application to Therapeutics,........ 269
Extirpation of Gland,........ 271

EDITORIAL.

Notes of Cases........ 274
Rubber Attachment,........ 276
An Extension Thimble,........ 277
Transactions of American Dental Association,........ 277

